# Mechanical and Antimicrobial Evaluation of Chitosan-Coated Elastomeric Orthodontic Modules

**DOI:** 10.3390/dj13100447

**Published:** 2025-09-29

**Authors:** Lucía Gabriela Beltrán-Novelo, Fernando Javier Aguilar-Pérez, Myriam Angélica De La Garza-Ramos, Arturo Abraham Cienfuegos-Sarmiento, José Rubén Herrera-Atoche, Martha Gabriela Chuc-Gamboa, Jacqueline Adelina Rodríguez-Chávez, Juan Valerio Cauich-Rodríguez

**Affiliations:** 1 Facultad de Odontología, Universidad Autónoma de Yucatan, Mérida 97000, Mexico; a11001445@alumnos.uady.mx (L.G.B.-N.); jose.herrera@correo.uady.mx (J.R.H.-A.); martha.chuc@correo.uady.mx (M.G.C.-G.); 2 Facultad de Odontología, Universidad Autónoma de Nuevo León, Monterrey 64460, Mexico; arturo.cienfuegossrm@uanl.edu.mx; 3 Centro Universitario de Ciencias de la Salud, Universidad de Guadalajara, Guadalajara 44340, Mexico; jacqueline.rchavez@academicos.udg.mx; 4 Unidad de Materiales, Centro de Investigación Científica de Yucatán, Calle 43 No. 130 x 32 y 34, Colonia Chuburná de Hidalgo, Mérida 97205, Mexico; jvcr@cicy.mx

**Keywords:** chitosan, glutaraldehyde, elastomeric modules, orthodontics, oral biofilm

## Abstract

**Background/Objectives**: Orthodontic appliances disrupt oral biofilm homeostasis, leading to an increase in plaque and disease risk. Elastomeric modules (EMs) promote bacterial growth due to their material composition. Surface coatings have been developed to reduce bacterial colonization. We evaluated the mechanical, antimicrobial, and cell viability properties of a chitosan coating for EMs. **Methods**: EMs were coated with chitosan (CS) and chitosan-glutaraldehyde (CS-GTA) to assess antimicrobial and cell viability. Uncoated EMs were used as a control. These surface-coated modules were characterized and analyzed with Fourier transform infrared (FTIR) and Raman spectroscopy, and tensile testing. Antibacterial activity was assessed by colony-forming units (CFU) counts after incubation. Cell viability was tested with gingival fibroblasts using the MTT assay. ANOVA, Tukey, Kolmogorov–Smirnov, and Kruskal–Wallis tests were used for statistical analysis. **Results**: Raman spectra of the chitosan coatings showed characteristic molecular vibration bands. ANOVA revealed a significant difference in mechanical properties between the materials and between the control and the CS-GTA groups, confirmed by the Tukey post hoc test. No significant difference was observed between the groups in the Yield Stress test. All the coated groups showed reduced CFU counts in the antibacterial assay. The average cell viability of the coated groups was 85% and 89%. **Conclusions**: We synthesized CS and GTA-cross-linked chitosan coatings. The coatings did not affect the mechanical properties of the elastomeric modules. The chitosan and glutaraldehyde-cross-linked CS coatings inhibited bacterial growth. No significant differences were observed in antibacterial activity between the CS and the GTA-crosslinked chitosan coatings.

## 1. Introduction

The oral biofilm is a complex community of microorganisms that adhere to and colonize the teeth and gums [1,2]. The oral microbiome maintains homeostasis in healthy individuals [3]; however, the placement of fixed orthodontic appliances disrupts homeostasis by creating larger retention zones, increasing dental plaque accumulation, and compromising oral hygiene [4]. As a result, homeostasis breaks down, and harmful pathogens grow [5]. These bacteria can cause tooth decay, early white spot lesions, and gum or periodontal disease [2,6]. The number of people undergoing orthodontic treatment has increased [7], and despite advances in orthodontic appliance design, maintaining good oral hygiene and implementing self-cleaning mechanisms remain challenging [4,6,8]. Tooth crowding, the most common malocclusion [9], adds a challenge to maintaining oral hygiene.

In orthodontics, elastomeric modules (EM) secure the braces to the archwire, generating the necessary force for tooth movement [10]. The main benefits of EMs are easy placement, more patient comfort, versatility, and low cost [11]. However, their main disadvantage is the easy adherence of microorganisms to their surface, which is attributed to the polyurethane, affecting the microbiome and homeostasis [12,13]. Jeon et al. aimed to reduce bacterial growth on elastomeric modules by adding chlorhexidine to exert a prolonged antimicrobial effect [14]. In another study, Hernández-Gomora et al. synthesized elastomers with silver nanoparticles with the same purpose [15].

Chitosan (CS), due to its biocompatibility, safety, biodegradability, and antimicrobial properties, is a widely used biopolymer with various dental applications [16,17,18]. CS is obtained from chitin, a structural component of the exoskeleton of crustaceans. Chitin is converted into CS through a deacetylation process in which the acetyl groups of the main chain are converted into amine groups [19,20,21,22,23]. The antimicrobial properties of CS are due to its positive cationic charge. When it interacts with the negative charge of the bacterial cell membrane, it breaks, releasing cell components [24,25]. D’Almeida et al. successfully demonstrated the antimicrobial effect of a chitosan coating on *E. coli* and *S. aureus* in a titanium alloy [26]. Similarly, Uysal et al. concluded that toothpaste containing chitosan, compared to non-fluoridated toothpaste, prevents enamel demineralization in patients undergoing orthodontic treatment [27].

Glutaraldehyde (GTA) is widely used for its disinfecting properties [28]. Its permanence aims to create a polymer with enhanced crosslinking to improve the material’s mechanical resistance while providing antimicrobial properties through its molecular structure [23,29]. GTA was included in this study because of its well-known disinfectant and antibacterial properties. It was also used for comparison to evaluate if the addition of GTA could enhance the material’s antibiofilm performance.

Cell viability assays ensure the safety of biomaterials in close contact with oral tissues such as the mucosa, gingiva, or dental structures. Any interaction between external materials and the oral environment can trigger an immune response [27]. The MTT assay is commonly used to assess cell viability [30]. Pathogenic biofilm formation remains a significant concern during orthodontic treatment with fixed appliances.

This study aimed to develop a chitosan coating for elastomeric modules, evaluate its antimicrobial and mechanical properties, and assess its effect on cell viability.

## 2. Materials and Methods

### 2.1. Chitosan-Coating of Elastomeric Modules

**Figure 1:**
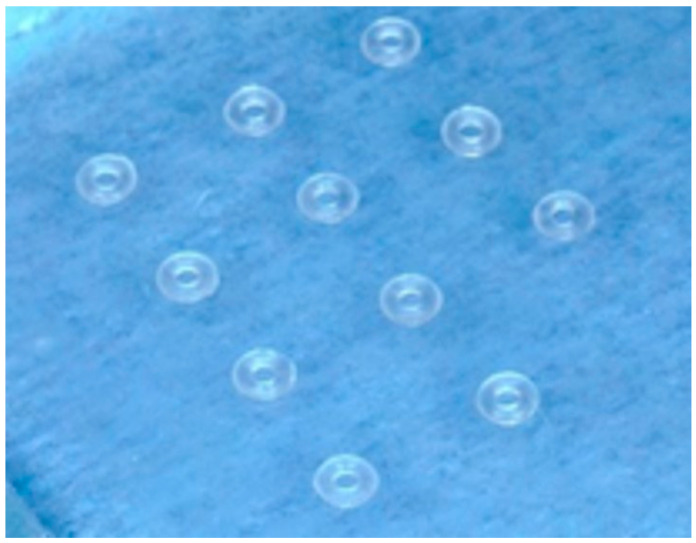
Chitosan-coated elastomeric modules.

Translucent elastomeric modules (EM) from TP Orthodontics (TP Orthodontics, Inc., La Porte, IN, USA) and chitosan (Sigma-Aldrich, St. Louis, MO, USA) with a molecular weight of 223.332 g/mol and 70–80% deacetylation were used. The first experimental solution was prepared by dissolving 100 mg of chitosan in 30 mL of a 0.4 M acetic acid solution at a pH of 4.5, which is equivalent to a 33.33% *w*/*v* solution. The solution was spin-coated at 450 RPM at room temperature for two hours. Afterward, the EMs were soaked in the CS solution for one minute and left to dry at room temperature for 120 h. Finally, they were neutralized with NaOH, washed with water, and dried for 24 h at room temperature. (Figure 1).

A second experimental solution was prepared using the same formula but with 75 mL grade II GTA at 25% (molar mass: 100.11 g/mol). Finally, the elastomeric modules coated with both solutions were neutralized with a 5% sodium hydroxide (NaOH) solution, washed with distilled water, and left to dry for 24 h. Uncoated elastomeric modules were used as a control group for comparison.

### 2.2. Physicochemical and Mechanical Characterization

The coated elastomeric modules were characterized using Fourier transform infrared attenuated total reflectance (FTIR-ATR) spectroscopy with a Nicolet 8700 spectrophotometer (Thermo Fisher Scientific Inc., Waltham, MA, USA). Spectra were acquired in a range of 4000 to 650 cm^−1^ using a zinc selenide crystal detector at a resolution of 4 cm^−1^ with an average of 50 scans.

Raman spectroscopy was performed with an InViaTM Raman Microscope from Renishaw (Wotton-under-Edge, Gloucestershire, UK) with a 633 nm laser at 50% power. The sample was analyzed in the spectral range of 100–3200 cm^−1^, with two accumulations, 1800 grating, a 50× objective, and a 10 s exposure time.

**Figure 2:**
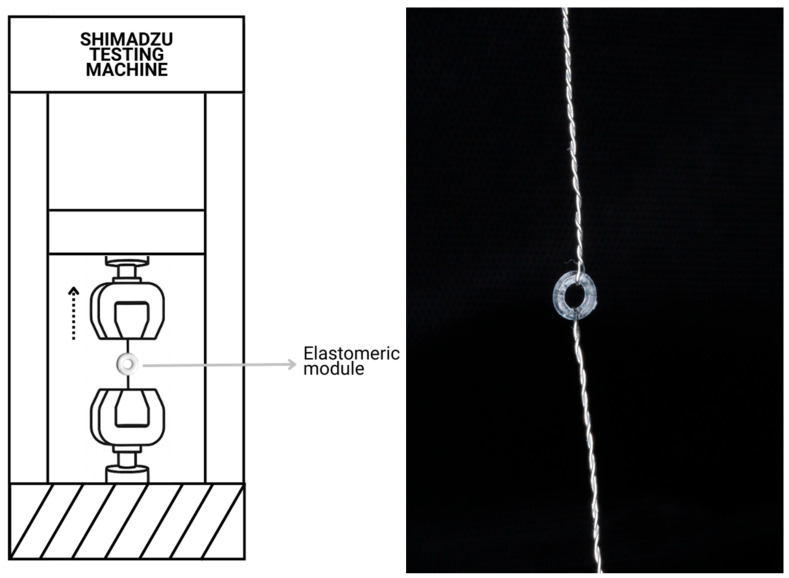
A diagram and photo of the testing apparatus using the 0.3 mm wire attachment.

A Mini Shimadzu universal testing machine (Shimadzu Corporation, Kyoto, Japan) was used to determine tensile strength. The tests were performed according to ASTM D624 standard guidelines, using a 1 kN load cell and a head travel speed of 10 mm/min. Due to the shape of the elastomeric modules, each EM was mounted on a specifically designed attachment made of 0.3 mm orthodontic stainless-steel wire, adapted to each end of the testing machine (Figure 2). The test was conducted and stopped when module breakage occurred. The obtained values were analyzed, and calculations were made for the following mechanical properties: yield deformation (YD), yield stress (YS), maximum deformation (MD), and maximum stress (MS).

### 2.3. Antibacterial Activity Assay

All procedures were performed aseptically in triplicate under a laminar flow hood (Labconco). The strains used were *Streptococcus mutans* ATCC au359 and *Streptococcus sobrinus* ATCC 27607, obtained from the Center for Research and Development in Health Sciences of the Autonomous University of Nuevo León (UANL) [31]. The bacteria were activated in Brain Heart Infusion (BHI) and trypticasein culture broth at 37 °C for 24 h according to the growth curve indicated by the supplier and adjusted to a bacterial density of 1 × 10^6^, corresponding to a McFarland standard of 0.0033. After activation, the bacterial solutions consisted of *S. mutans*, *S. sobrinus*, and a mixture of *S. mutans* and *S. sobrinus*. Each 100 µL solution was deposited in Eppendorf tubes. Then, three elastomeric modules of each coating were placed in each bacterial solution in each Eppendorf tube and incubated for 24 h at 37 °C. Each module from every group was subsequently removed from each bacterial solution and placed in a new Eppendorf tube containing 1 mL of sterile water. The tubes were then vortexed for 20 s and processed for serial dilution. An aliquot of 100 µL was taken from the fifth dilution and inoculated onto Petri dishes containing Miti salivarius agar (MSA). The Petri dishes were then incubated for 24 h at 37 °C, and colony-forming units (CFU) were counted.

### 2.4. Cell Viability

In the cell viability tests, the CS- and CS-GTA-coated modules were placed directly in wells and incubated for 24 h. The MTT technique (100 µL of MTT at a concentration of 0.25 mg/mL in cell culture medium (DMEM) and incubation of the plates for 4 h under standard conditions) was performed using ATCC PCS-201-018 gingival fibroblasts [31]. A density of 10,000 cells per well was placed in a volume of 100 µL per well. After 24 h of incubation, 100 µL of dimethyl sulfoxide was added to solubilize the formazan salts.

The culture plates were read in an iMark Bio-Rad microplate reader (Bio-Rad Laboratories, Inc., Hercules, CA, USA) (LEM-01) at 570 nm. Uncoated modules served as the positive control, and 0.12% chlorhexidine gluconate (CHX) was the negative control. Soluble MTT reduction was used to determine fibroblast cell viability. The experiments were conducted in quintuplicate, and the Kolmogorov–Smirnov and Kruskal–Wallis tests were used to analyze the results.

### 2.5. Statistical Analysis

One-way analysis of variance (ANOVA) was performed on the mechanical properties and antibacterial evaluation using Jamovi software (2.6.44 desktop version). The Tukey post hoc test was used to compare the study groups. The Kolmogorov–Smirnov and Kruskal–Wallis tests were used for the cell viability assay. A *p*-value < 0.05 was considered significant in all statistical tests.

## 3. Results

### 3.1. Physicochemical and Mechanical Characterization

**Figure 3:**
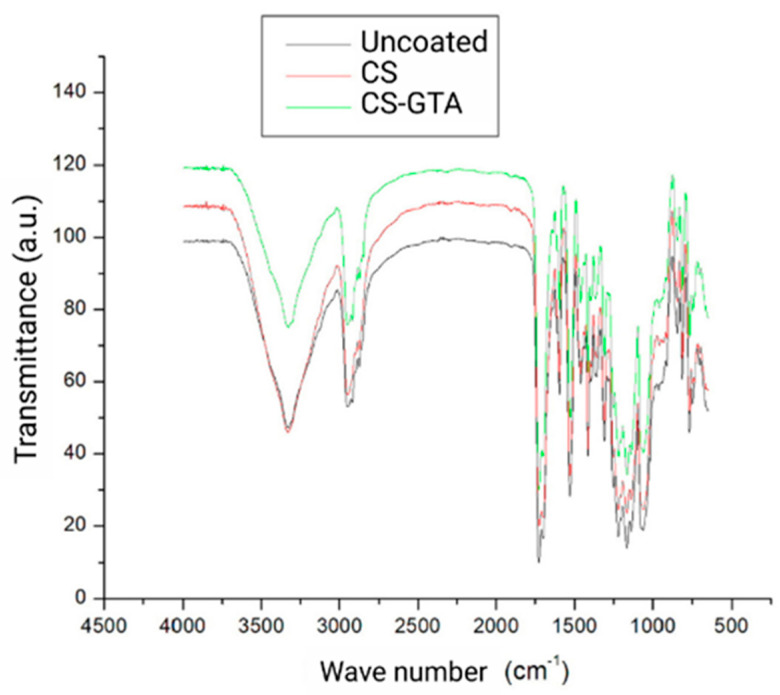
FTIR spectrum of the elastomeric modules; CS = chitosan; GTA = glutaraldehyde; Control, uncoated.

The FTIR spectra were obtained from the modules with the experimental coatings, CS and CS-GTA, and the control group, which did not have a coating (Figure 3). The FTIR spectra of CS and CS-GTA exhibited a band at 3332 cm^−1^, corresponding to the stretching of –NH_2_ and O–H groups. Likewise, the bonds found at 2873 and 2954 cm^−1^ are attributed to stretching between C and H. The band found at 1595 cm^−1^ comes from C=O, and the one at 1061 cm^−1^ is assigned to the stretching of C–N. A band was detected at 1414 cm^−1^, corresponding to the stretching vibration of the C–N bond in the amide II mode. The peak at 3334 cm^−1^ is characteristic of stretching with the O–H and N–H bonds of the alkyl group (CH). The degree of deacetylation can be observed at 1309 and 1725 cm^−1^. The band at 1596 cm^−1^ is attributed to the stretching of amide I, which arises from a non-deacetylated residue. A peak at 1413 cm^−1^ was observed corresponding to the O–H vibration of amide I, and the band at 1361 cm^−1^ to the CH_3_ group. The band identified at 1462 cm^−1^ is characteristic of the C–N stretching in the amide group II. The bands between 1530 and 815 cm^−1^ are part of chitosan’s C–O stretching. There were no substantial changes in the bands identified between pure CS and the crosslinking agent (GTA). However, certain variations were observed at 2918 and 2951 cm^−1,^ as they presented shifts to 2925 and 2945 cm^−1^, respectively. These changes can be attributed to the symmetrical stretching of the CH_3_ group. In the spectra of the uncoated modules, two peaks were observed at 962 and 1394 cm^−1^; these bonds were not identified in the experimental coatings analysis and corresponded to the symmetrical stretch of CH_3_.

**Figure 4:**
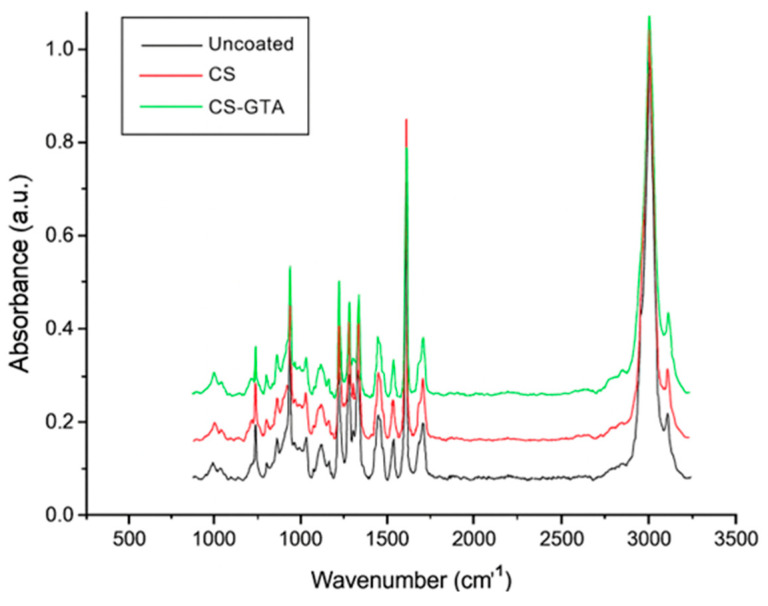
Raman spectroscopies of elastomeric module coatings; CS = chitosan; GTA = glutaraldehyde; Control, uncoated.

Raman spectra are shown in Figure 4. The chitosan coating exhibited a 2925 cm^−1^ band corresponding to the stretching vibration of v(CH_2_). Likewise, the band at 1616 cm^−1^, attributed to the bending vibration in the δ(NH_2_) plane, was found. Other bands identified were 865 and 1184 cm^−1^, which are characteristic of the C–C–O stretching vibration. Regarding the spectroscopy of CS crosslinked with GTA coatings, a band is observed at 638 cm^−1^. The Raman shift at 638 cm^−1^ is typically associated with C–C–C skeletal bending or deformation modes of the aliphatic chain present in the glutaraldehyde structure. The band observed at 1729 cm^−1^ is attributed to C=O, and the one at 1616 cm^−1^ concerns the C=N stretching. Finally, the peak at 1537 cm^−1^ is attributed to the C=C vibration.

### 3.2. Mechanical Characterization

**Figure 5:**
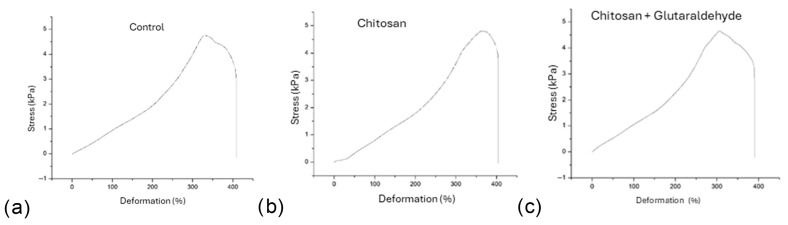
Representative strain–stress curves from the tensile mechanical test of the elastomeric modules: (**a**) uncoated, (**b**) CS-coated, and (**c**) CS-GTA-coated.

The mechanical evaluation of coated elastomeric modules is shown in Table 1 and Figure 5. The yield strain (YS) showed an average of 336 ± 14.4% for the control group, 324 ± 18% for CS, and 314 ± 11.3% for CS-GTA. The ANOVA test showed a significant difference between the materials. Specifically, in the Tukey post hoc test for the group evaluation, a significant difference was found only between the control group and the CS-GTA group.

Regarding the Yield Stress (σ_Y_), the control group obtained an average of 5.06 ± 0.31 kPa. The CS coating and the crosslink with GTA had values of 4.93 ± 0.26 and 4.74 ± 0.22 kPa, respectively. An ANOVA with the Tukey test was performed. No significant difference was found between the groups.

In the analysis of maximum deformation (MD), the averages were 409 ± 11.9% for the modules without a coating, 398 ± 4.1% for the CS group, and 393 ± 18.5% for the CS-GTA group. The ANOVA showed no significant difference between the groups. Finally, regarding the maximum stress (σ_max_) results, the control group average was 5.12 ± 0.28 kPa, while the CS coatings and CS-GTA had averages of 4.94 ± 0.26 and 4.82 ± 0.24 kPa, respectively. The ANOVA showed no significant differences. However, the Tukey test did show a difference between the control and crosslinked groups.

### 3.3. Antibacterial Activity Assay

**Figure 6:**
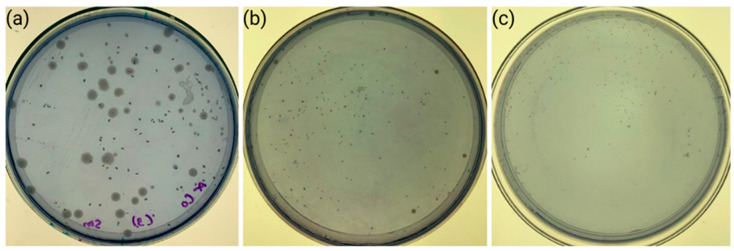
Petri dishes show the antibacterial effect of the (**a**) control, (**b**) chitosan, and (**c**) chitosan-glutaraldehyde.

The coated modules across all bacterial compositions demonstrated a reduction in CFUs (Figure 6). The ANOVA statistical test showed a significant difference for all three bacterial compositions. The post hoc Tukey test revealed statistical differences between the groups in the reduction in CFUs. No statistical differences were found in any bacterial composition between the CS-coated and the CS-GTA-coated groups. The lowest bacterial growth was for the CS-GTA-coated module with the *S. mutans* + *S. sobrinus* combination, followed by the CS-coated module with the *S. mutans* solution. The CS and CS-GTA coatings exhibited similar antimicrobial activity, which was significantly lower than that of the uncoated modules against all bacterial compositions (Table 2).

### 3.4. Cell Viability

**Figure 7:**
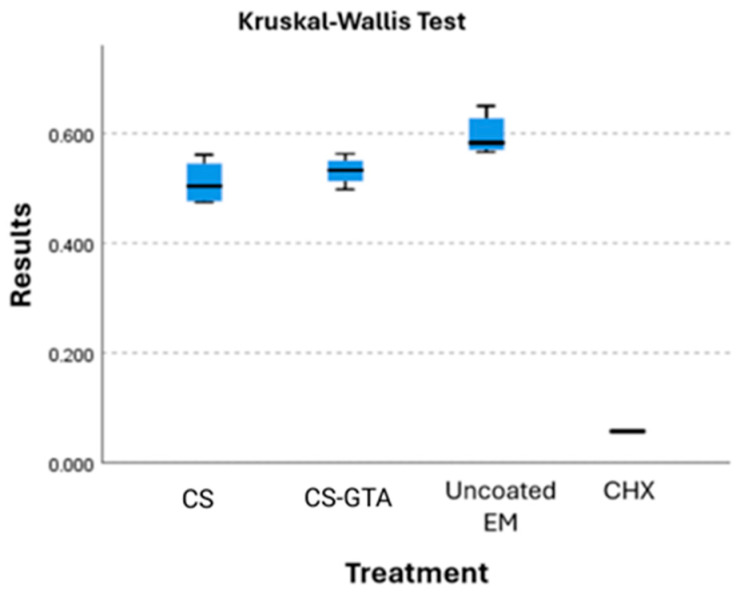
Cell viability of coated and uncoated elastomeric modules.

The average absorbance of the uncoated modules was set as 100% cell viability. The average cell viability of the coated groups, consisting of CS and CS crosslinked with GTA, was 85% and 89%, respectively (Figure 7). The Kruskal–Wallis test compared the four groups since the data did not follow a normal distribution. A statistically significant difference was found between the groups. Pairwise comparisons were subsequently conducted using the Mann–Whitney U test. The results showed that only the comparison between the chlorhexidine group and the uncoated modules remained statistically significant. No significant differences were found between the other groups after correction for multiple comparisons. When comparing CS with CS-GTA, and CS-GTA against the uncoated group, no significant difference was found. However, a significant difference in cell viability was found between the CS group and the uncoated group. Figure 6 visually shows this difference.

## 4. Discussion

Infrared spectroscopy confirmed the coating of the elastomeric modules in the CS and CS-GTA groups. This outcome concurs with Li et al. [20]. As Uysal et al. mentioned, the identifiable changes in the FTIR spectra occurred between 3200 and 3600 cm^−1^ [27]. Adding GTA as a crosslinking agent produces identifiable changes [32,33]. Conversely, the results of Monteiro et al. differ when analyzing CS-GTA since they did not observe any significant changes [34]. The bands identified at 1309 and 1725 cm^−1^ in this study regarding the degree of deacetylation correspond with findings by Beppu et al. and Jeon et. al [35,36]. The degree of deacetylation plays an important role because it modifies the properties and behavior of CS [37]. Various bands identified from the experimental coatings matched the investigations of Cusihuamán et al. These vibrations ranged between 3400 and 3200 cm^−1^ for CS, corresponding to the stretching of the O–H and N–H bonds [21,23,33]. Escobar-Sierra et al. concluded that the bond at 2947 cm^−1^ is from the alkyl group (CH) [37]. Other infrared analyses of CS have reported that a range between 1400 and 1600 cm^−1^ corresponds to the group of primary and secondary amides found in the coatings analyzed in this study [33,35,38].

Regarding Raman spectroscopy, Ren et al. found characteristic C-H bonds at 895 and 1146 cm^−1^ [39]. These results are equivalent to the peaks identified at 865 and 1184 cm^−1^, which are characteristic of the C-C-O stretching vibration, as reported by Gamboa-Solana [33]. On the other hand, Mai et al. found the main CS signal at 680 cm^−1^, which coincides with the bond found at 693 cm^−1^ [40]. Several studies have discussed the properties of elastomers in dentistry and orthodontics, as well as efforts to develop antimicrobial properties. Berni Osorio et al. subjected elastomeric ligatures to different disinfectant solutions and concluded that 2% glutaraldehyde does not alter their mechanical properties [41], in contrast to our study. Properties were modified when crosslinked with GTA, where a difference was observed compared to the control group [42]. Melo-Pithon et al. compared various sterilization methods on elastic chains, including alcohol, autoclave, ultraviolet, peracetic acid, and glutaraldehyde. Their results indicated that ultraviolet light was the least efficient [43]. However, immersion in different solutions did not affect the mechanical properties of the material. Stevenson et al. [44] indicated that to produce a significant change in the mechanical properties of elastomers, acidity, oxygen, and temperature must be modified. This finding could be a potential explanation why immersion in the acidic medium (glutaraldehyde) may have altered the material’s properties, thereby leading to the observed results.

Immersion in the acidic medium containing GTA reduced the elastomeric properties at the yield stress (YS) parameter. Still, the overall mechanical performance of the elastomeric modules (EMs) remained unaffected. Similar findings were reported by Losito et al., who observed no significant differences in the mechanical properties of elastomeric materials after immersion in chitosan or peracetic acid solutions [45]. In another study, Evangelista et al. demonstrated that the mechanical properties of the modules are negatively affected when exposed to disinfectant liquids for more than one hour [10]. Likewise, Terheyden et al. analyzed various sterilization techniques for maxillomandibular immobilization ligatures, demonstrating that polyurethane was the most resistant and that sterilization with ethylene oxide was the most efficient [46]. Otherwise, the analysis of the maximum deformation yielded similar results to those of Jeon et al., who also found no observable differences [4]. Alternatively, McKamey et al. [47] demonstrated that a chlorine-substituted poly(para-xylylene) coating on orthodontic elastic chain modules improves the material’s mechanical properties. Numerous authors have noted that the mechanical properties of elastomeric ligatures depend on their color [8,11,48,49]. This fact is the reason why transparent modules were used in this research, which is a limitation in this study.

Furthermore, the mechanical properties of ligatures change when exposed to a moist environment. According to Halimi et al., elastomeric modules not exposed to artificial saliva presented different mechanical properties [50]. Mechanical degradation over time also plays an important role in the mechanical capacity of elastomers [51]. Considering that the change of modules occurs every four weeks, it is important to consider whether the properties may be affected in the long term for future research [52].

It is relevant to clarify that isolated bacteria were used, and no significant differences were detected in bacterial growth. Kamarudin et al. conducted research using a similar methodology and demonstrated the efficiency of elastomers with prolonged release of a chlorhexidine (CHX) coating [53]. Another comparable study with favorable results demonstrated the antimicrobial efficacy of CHX as an elastomer coating without compromising its mechanical properties [14]. On the other hand, Uysal et al. demonstrated the effectiveness of using chitosan in the oral cavity as an antimicrobial agent added to toothpaste to reduce white spot lesions in patients wearing braces [27].

The literature reports variable outcomes, in line with the present study, when evaluating different antimicrobial agents. For instance, Benson et al. [54] analyzed the effect of fluoridated elastomeric ligatures but found them ineffective in reducing the growth of oral bacteria. Similarly, Doherty et al. concluded that fluoride-releasing modules with prolonged release do not offer any anticariogenic benefits [55]. Another study investigated the use of silver-coated elastomers as a strategy to achieve antimicrobial effects; however, no significant differences were observed [56]. In contrast to these previous approaches, the methodology in this study successfully synthesized chitosan coatings on elastomeric modules. Nevertheless, limitations remain, particularly regarding the long-term stability and sustained effectiveness of the coating, which warrants further investigation.

Adding a coating to the modules represents only one of the many factors that can affect their mechanical capacities.

Our findings regarding cell viability are relevant because they confirm the biocompatibility of the coatings. Moreover, the crosslinked group showed slightly better cell viability compared to the CS-only coating.

The inoculation of two or more strains identified from the oral biofilm creates an in vitro environment that is closer to the natural conditions in which bacteria normally develop. Sharma et al. determined that the color of elastomeric ligatures intervenes in microbial adhesion [8]. Additionally, Shi et al. demonstrated that chitosan is an efficient scaffold in drug-controlled release [57]. Garner et al. verified the effectiveness of chitosan nanoparticles as a silicone coating against *C. albicans* [58]. The literature reports similar results from the bacteriological test by Padois et al., who successfully manufactured orthodontic elastic polyurethane chains with a CH-loaded layer [59]. D’almeida et al. obtained similar information when analyzing the antibacterial action against *S. epidermis* [26]. Specifically, Sarasam et al. aimed to develop efficient chitosan matrices that inhibit the growth of oral pathogens [60]. Favorable results were found in reducing *S. mutans* growth but not *A. actinomycetemcomitans*.

Nevertheless, different results have been reported with various antimicrobial agents, as noted by Doherty et al., since modules with prolonged fluoride release do not provide any anticariogenic benefit [55]. Kim et al. found no significant differences in bacterial development in silver-coated elastomers [56]. Bacteria associated with dental caries may respond differently and independently from each other.

Some cytotoxicity effects of chitosan have been well documented. Frigaard et al. demonstrated the low cytotoxicity of chitosan nanoparticles [61]. Research by Raviña et al. found that hyaluronic acid with chitosan-g-poly(ethylene glycol) nanoparticles efficiently delivered different types of gene molecules [62]. Safe human areas have been identified for the application of chitosan nanoparticles, with the oral cavity being one of them. Furthermore, chitosan nanoparticles exhibit a cytotoxic effect on cancerous cells without harming normal cells. pH appears to influence chitosan cytotoxicity, but this requires further research [63].

Additionally, in vitro cytotoxicity assays using the MTT method with chitosan and its derivatives have demonstrated lower toxicity to breast cancer cells [64,65]. These studies agree on the low toxicity of chitosan compared to other antimicrobial substances, like chlorhexidine (CHX). CHX is a widely used antiseptic in the medical field and dentistry. However, its cytotoxic effects have been proven in human fibroblasts, myoblasts, and osteoblasts in vitro [66,67].

Finally, according to ISO 10993-5 standards, materials are considered non-cytotoxic when cell viability exceeds 70% [68]. In this study, the chitosan-based coatings demonstrated cell viability values over 85%, which fall within the acceptable range for biocompatible materials. Therefore, these results suggest that the proposed coatings are biocompatible for potential intraoral applications.

## 5. Conclusions

CS and GTA-cross-linked CS coatings were successfully synthesized without compromising the mechanical properties of the elastomeric modules. Although a slight reduction in yield stress was observed with the addition of the crosslinking agent, the coatings maintained adequate mechanical performance.

It is important to note that other factors influencing the mechanical behavior of elastomeric materials—such as long-term performance of the coatings, humidity, temperature, pH, and color changes in the elastomeric module—were beyond the scope of this study and should be addressed in future investigations.

In terms of antimicrobial activity, both CS and CS-GTA coatings significantly inhibit the growth of *S. mutans*, *S. sobrinus*, and their combination. No significant differences were observed between the two experimental coatings. Moreover, this research focused on bacterial strains recognized as primary contributors to caries formation; future studies should consider evaluating additional bacterial species to validate these findings.

Finally, the two experimental coatings demonstrated acceptable cell viability and, compared to CHX, exhibited excellent antimicrobial properties while maintaining biocompatibility. These results suggest that chitosan-based coatings represent a promising strategy for reducing bacterial colonization on orthodontic elastomeric modules without adversely affecting their mechanical integrity.

## Figures and Tables

**Table 1:** Tensile mechanical properties of the elastomeric modules.

Material	YS * (%)		σ_Y_ (MPa)	MD (%)	σ_max_ (MPa)
Uncoated	336 ± 14.4	^a^	5.06 ± 0.31	409 ± 11.9	5.12 ± 0.28
CS	324 ± 18	^ab^	4.93 ± 0.26	398 ± 14.1	4.94 ± 0.26
CS-GTA	314 ± 11.3	^b^	4.74 ± 0.22	393 ± 18.5	4.82 ± 0.24

CS = Chitosan; GTA = glutaraldehyde. Groups that do not share letters are statistically different from each other. YS = Yield Strain, σ_Y_ = Yield Stress, MD = Maximum Deformation, σ_max_ = Maximum Stress. * *p*-value < 0.05. Different lower case letters indicate a significant difference among groups.

**Table 2:** CFU values and standard deviations of the experimental coatings.

Experimental Coatings	*S. mutans*	*S. sobrinus*	*S. sobrinus +* *S. mutans*
Ch			
Mean	208	906.666667	1544
SD	60.39867548	154.108187	421.729771
CS-GTA			
Mean	645.3333333	528	316
SD	184.2751566	276.781502	150.359569
Control			
Mean	733.3333333	1116	1368.333333
SD	110.01	86.0697392	165.9889555

## Data Availability

The data that support the findings of this study are available from the corresponding author upon reasonable request.
